# Effect of Extruder Type in the Interface of PLA Layers in FDM Printers: Filament Extruder Versus Direct Pellet Extruder

**DOI:** 10.3390/polym15092019

**Published:** 2023-04-24

**Authors:** Antoni Pagés-Llobet, Francesc X. Espinach, Fernando Julián, Helena Oliver-Ortega, José Alberto Méndez

**Affiliations:** 1 LEPAMAP-PRODIS Research Group, Department of Chemical Engineering, Agriculture and Food Technology, Universitat de Girona, 17003 Girona, Spain; antoni.pages@udg.edu (A.P.-L.); francisco.espinach@udg.edu (F.X.E.); fernando.julian@udg.edu (F.J.); jalberto.mendez@udg.edu (J.A.M.); 2 Department of Materials Science and Engineering, Universitat Politècnica de Catalunya, Colom 1, 08222 Terrassa, Spain

**Keywords:** additive manufacturing, FDM (Fused Deposition Modeling), Poly(lactic acid), mechanical properties, interface, cristallinity

## Abstract

FDM (Fused Deposition Modeling) is one of the most used and industrially applied additive manufacturing processes due to its fast prototyping and manufacturing, simplicity, and low cost of the equipment. However, the mechanical properties of the printed products have a large dependence on orientation and interface strength between layers which is mainly related to the thermal union obtained. This thermal union has a large dependence on the melting and cooling down process. Additionally, the materials used must be extruded in a continuous filament before their use, which limits the materials used. However, a pellet extruder could be used directly in the printing equipment, avoiding filament extrusion. In this work, specimens of PLA (Poly(lactic acid)) with different bead orientations have been produced via filament or pellet extrusion to compare the effect of the different melting processes in the manufacturing methodology. Pellet extruded specimens showed higher infill and mechanical properties. These results were related to better adhesion between layers due to the longer melting and cooling process. The result was confirmed using DSC and XRD techniques, where a higher crystallinity was observed. A bicomponent specimen (50% pellet–50% filament) was prepared and tested, showing higher mechanical results than expected, which was, again, due to the better thermal union obtained in the pellet extruder.

## 1. Introduction

Fused deposition modeling (FDM) is a rapid prototyping and rapid manufacturing technique inside the so-called additive manufacturing processes [1]. FDM has shown a noticeable increase in popularity due to its simplicity, cost, and the appearance of RepRap 3D printers [2]. One of its characteristics is the ability to build prototypes with different mechanical properties depending on the strategy used to build the layers, the orientation of the beads, the filling percentage, the diameter of the extruder, and the thickness of the layer when using the same material [3,4,5]. Thus, the mechanical properties of specimens manufactured with FDM cannot be based on the properties of the base material, as such specimens have to be explored as structures. Therefore, fundamental properties such as Young’s modulus can differ between specimens based on the same material when the building parameters of such specimens vary [6,7]. 

FDM-obtained specimens show an internal structure that defines the mechanical properties of such specimens [8]. Polymer beads are extruded and form a contact zone between the beads in the same layers and the beads in the layer below. Therefore, the strength of the specimen is defined by the strength of the beads and the strength of the interface between the layers and the beads in the same layer. The strength of the beads is defined by the mechanical properties of the polymer and its orientation against the loads, and the strength of the interface is defined by the quality of the thermal union between beads and their area [9,10,11]. The interfacial area changes with the orientation of the beads and the percentage of filling of the specimen. PLA (Poly(lactic acid)) has been largely studied in the literature as one of the most promising biopolymers for FDM [12]. Nonetheless, PLA is commonly additivated for FDM, which could compromise some of its properties [13]. Studies have been carried out with orientations parallel or transversally to the printer bed, filling percentages, filling patterns, etc. [14,15,16,17]. The mechanical properties of the printed specimens showed a dispersity of results, ranging between 30 and 60 MPa [18,19]. Authors generally agree on the necessity to heat the printer bed to temperatures close to the glass transition temperature (T_g_) of PLA, around 60 °C [20]. To improve the quality of the interface, some post-treatments could be applied, such as Bsuch thermal or chemical [20,21]. It has been demonstrated that crystallinity has a high influence on the final properties of the 3D-printed part [11,22]. In some cases, post-treatment methodologies are carried out to modify crystallinity and, as a consequence, the mechanical properties of polymers [10,23]. Another alternative is the use of micro or nanofillers, which lead to enhanced mechanical properties. Nevertheless, the orientation, dispersion, and aspect relation of the fillers become another parameter that influences the mechanical performance of these structures [24]. However, despite the improvements to these fillers, one of the main drawbacks is the production of the filament [25]. The filament must have a regular thickness and shape without any voids or defects. 

Traditionally, FDM materials have the shape of a continuous filament that feeds the equipment extruder. Nonetheless, the catalog of commercial FDM filaments is less extensive than the available polymers in the shape of pellets [26]. Indeed, it is possible to obtain filament from such pellets, but it is necessary for the extrusion equipment to be capable of delivering filament with the required uniformity in its diameter [27]. This technology is not as widely known as filament extrusion but has the advantage of having a lower cost and the possibility of using commercial materials not available as filament [28]. This is known as pellet-based extrusion or fused granular fabrication (FGF). These printers have a larger extruder that allows the melting of pellets, while the manufacturing technique is the same: deposition of melted filaments [29]. This technique could reduce the cost while improving the efficiency of the FDM process. Therefore, it is expected to increase the use of different types of composites and nanocomposite materials for the production of the filament, and its associated difficulties are avoided [30,31]. Concerning the mechanical properties in the literature, some studies have compared the properties of specimens obtained with FDM and FGF. Reddy, B.V. et al. studied the impact of nozzle and chamber temperatures, bead distance, and bead intersection on the bead-to-bead strength [32]. The researchers found that the strength of the bead-to-bead interface increased with the chamber temperature and the bead-to-bead distance. In the same paper, the researchers stated that the strength of the interface between beads printed with FGF was higher than those printed with FDM. This paper referred to beads printed in the direction perpendicular to the loads applied during tensile tests. In a similar study, Alexandre et al. studied the processability, economy, and mechanical properties of FGF and FDM PLA obtained specimens [33]. The authors found that it was possible to obtain slightly higher mechanical properties with FGF than with FDM while reducing the printing costs and the time needed to print the same piece. The authors of the paper agreed on the necessity of further research to analyze how process parameters affect the geometric performance of printed parts. Singamnemni et al. devoted a study to the parameters affecting the mechanical properties of PLA-based composite specimens obtained with FGF and compared the results with mold-injected specimens [34]. The authors used a spiral path to obtain the specimens and found that the processing conditions that returned the highest mechanical properties were a print speed of 30 mms, a printing temperature of 200 °C, and a temperature of the printing table of 160 °C. The use of a material with more than one phase adds interest to the study but restricts the results to such a material. A recent study by Fontana, L. et al. researched the tensile properties of PLA specimens obtained with FGF and with different bead orientation strategies [35]. The authors found that infill percentage had a noticeable impact on tensile properties and the maximum tensile stress was obtained with a 75% infill strategy. The authors did not use a 100% infill strategy. Nonetheless, FGF is an incipient technique, and more studies are necessary to assess the parameters affecting this technology. 

This work aims to contribute to the development of pellet-based extruders and analyze the effect of the different melting procedures. Standard tensile and flexural specimens were printed in a 3D printing machine with a filament and a pellet extruder at different orientations to analyze the effect of the extruder and the melting system. In this case, the polymer and the printing conditions (speed, infill, nozzle temperature, bed temperature, etc.) were the same, and the extruder and the melting process was the main parameter. The influence of the orientation of the layer to the bed printer was analyzed, as the mechanical dependency on it is well-known. Moreover, a dual specimen was produced to assess the viability of these materials. 

## 2. Materials and Methods

### 2.1. Materials

Poly(lactic acid) (PLA) from Total Energies-Corbion (Gorinchem, The Netherlands) trade Luminy L105, an injection grade, was used as the polymer for the filament extrusion directly in its pellet form for the pellet extruder of the 3D printer. 

### 2.2. Methods

#### 2.2.1. PLA Filament Extrusion

**Figure 1:**
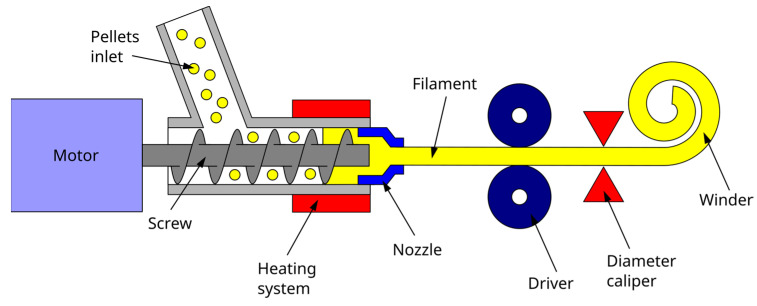
Filament production from the pellet material in the filament extruder equipment.

The filament was extruded with a3devo Next 1.0—Advanced extruder (Atoomweg, The Netherlands). This equipment has a hopper that feeds the pellet extruder with the material in question through a worm. Previous to its use, pellets are dried in an oven at 100 °C for 4 h. The pellets pass through four zones with different temperatures higher than the melting temperature of the polymer. Next, the material in a viscous state leaves the filter and is driven between two bearings that stretch the material, which, using a thickness control, regulates the diameter of the filament. This thickness is determined in the “material configuration” menu available on the extruder and was established at 1.75 mm. A simplified diagram of the extrusion process is shown in Figure 1. Having selected the material and the thickness, the machine has preset parameters, such as spindle speed and temperatures, which can be manipulated according to the technician’s discretion. Once the thickness of the filament is stabilized with a tolerance of ±0.10 mm, the filament is positioned on the coil, and winding begins. Once the filament is produced, it is stored in a desiccator at room temperature until its use. 

#### 2.2.2. Specimen Obtention

**Figure 2:**
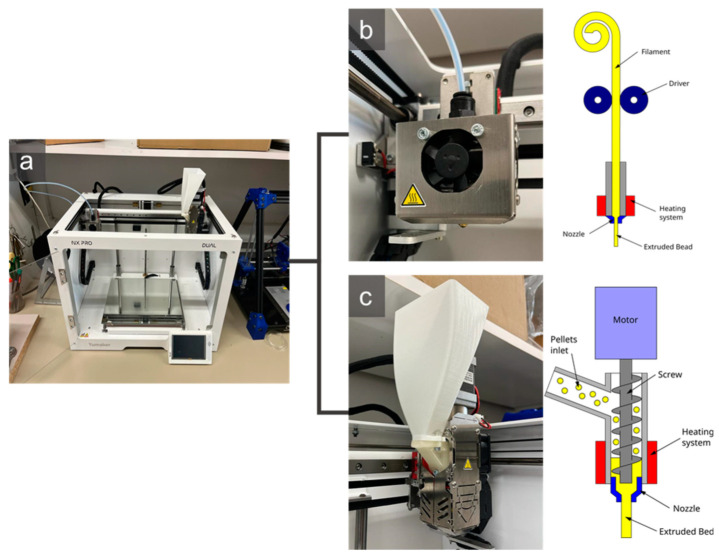
(**a**) The 3D printer used to manufacture the specimens with the two different nozzles; (**b**) Filament extruder; (**c**) Pellet extruder and its schematic representation.

The 3D models of the standard specimens were modeled with SolidWorks^®^. The models were saved in STL format and processed into G code with Simplify3D provided by the FDM equipment distributor. The specimens were obtained with an FDM printer 3D NX PRO DUAL by Tumaker (Guipuzkoa, Spain). This equipment has two independent nozzles, one fed with the filament (diameter: 0.4 mm) and the other with pellets (diameter 0.8 mm) (Figure 2). These nozzles were used to obtain 6 specimen sets for different internal bead orientations regarding the X-Y planes. The parameters used with both nozzles were:

- Speed: 27 mm/s;
- Bed temperature: 55 °C;
- Output temperature: 200 °C;
- Inlet temperature (pellet): 160 °C;
- Filling percentage: 100%;
- Cooling speed: 90%;
- Layer height 0.2 mm.

**Figure 3:**
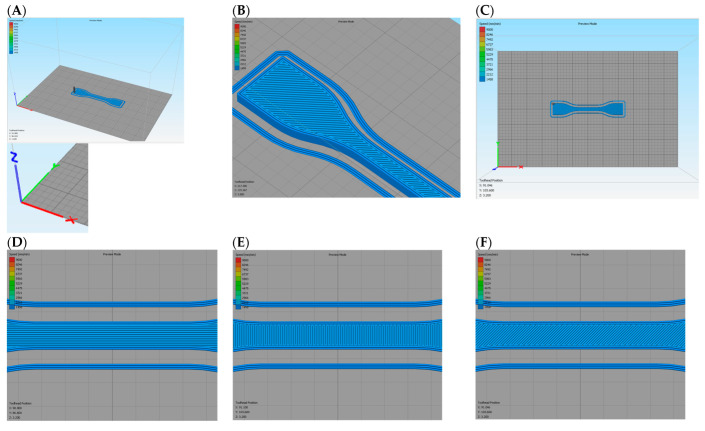
(**A**) Main axis orientation concerning the printing table and position of the specimens in the table; (**B**) Detail of the clipping area of a specimen printed at 45° bead orientation; (**C**) View of the specimen to the printing table with X (green axis) and Y (red axis) axis coinciding with the view; (**D**). Detail of a 0° bead orientation; (**E**) Detail of a 90° bead orientation; (**F**) Detail of a 45° bead orientation.

The orientation of the specimens regarding the printing table were 0, 45, 90, and 90/0°. For the specimens prepared in the 3D extruder, PLA was dried previous to its use at 100 °C for 4 h. Figure 3 shows the relative orientation of the beads with respect to the printing table. 

#### 2.2.3. Infilling Percentage of the Specimens

The density of the specimens was obtained with an ACS 220-4 balance from Kern (Balingen, Germany) with an error of ±0.1 mg. To carry out this test, the samples were first conditioned in a vacuum line at room temperature for two hours to extract the moisture. Finally, the test specimens were removed from the chamber and weighed on the balance. It should be noted that the volume of the pieces was known since the measurements were taken physically. We refer to this as real volume (V_R_). For the bending test specimens being a rectangular structure:

(1) $$V_{R}=a\cdot p\cdot h$$

where (*a*) is the width of the test tube, (*p*) is the depth, and (*h*) is its height. On the other hand, for the tensile test specimens, due to their geometry, the volume was obtained from SolidWorks. The density of the materials ($\rho_{s}$) and the weight of the specimens (*m*) were known and the volume ($V_{A}$) was obtained as expressed by the following equation:

(2) $$V_{A}=\frac{m}{\rho_{s}}$$

*V_A_* is the apparent volume of the structure. From the real and apparent volume, the empty volume (*V_B_*) in the sample was calculated as:

(3) $$V_{B}=V_{R}-V_{A},$$

From the empty volume, the percentage of apparent filling and density of the samples ($\rho_{A}$) was obtained as expressed by the following equation:

(4) $$\rho_{A}=\left(1-\frac{V_{R}-V_{A}}{V_{A}}\right)\times 100,$$

#### 2.2.4. Evaluation of the Mechanical Properties

Tensile tests were conducted with a Universal testing machine, DTC-10, IDM test (Donostia, Spain). The tensile test was carried out with standardized mold injected and 3D printed test specimens type IV, following ASTM D638. At least five specimens of all the configurations were tested. The orientation of the printed specimens during the tensile test was the same as the printing layer. The test was carried out in the X plane. 

Flexural properties were measured with the same equipment with a three-point bending configuration, following UNE-EN-ISO 178. In this case, the force was applied in the Z plane, perpendicular to the orientation of the deposited layers. 

#### 2.2.5. Thermal Transitions and Crystallinity

The first melting behavior of the PLA specimens was studied using Differential Scanning Calorimetry (DSC) to analyze the cooling behavior and differences in the thermal transitions and crystallinities. The scans were performed with a TA instrument Q2000 working from 40 to 200 °C with a heating range of 10 °C/min and under an inert atmosphere of N_2_ with a constant flow of 40 mL/min. The crystallinity was corroborated using X-ray Diffraction (XRD) in a D8 QUEST ECO (Bruker, Madrid, Spain) with a Cu-Kα radiation (λ = 0.15406 nm). The examined 2θ range was from 1.2 to 30° with a working range of 40 KV and 40 mA. 

#### 2.2.6. Optical Microscope Analysis

Optical pictures of the specimens and their fracture after flexural test were performed with two different optical microscopes: a digital microscope, Jiusion 40 A 1000, with a resolution of 40 to 1000× and a polarized optical microscope, NIKON SMZ1000, with a resolution of 8–80×. In both microscopes, the photographs were obtained from the external layers of the printed specimens. These layers correspond to the X-Y plane. 

## 3. Results

The preparation of the filament from the commercial injection-molding degree was complicated due to the lack of additives in the PLA. The filament obtained was fragile and sometimes collapsed during the printing process. Nonetheless, the diameter obtained was kept constant, close to the 1.75 mm fixed on the diameter caliper. The fragility of the PLA filament is directly associated with the intrinsic properties of the PLA and the lack of additives used in commercial PLA filaments. However, avoiding the use of these materials does not compromise the biodegradable behavior of the polymer and reduces the costs.

On the other hand, pellet specimens were directly obtained. The main difficulty observed during the printing process was the requirement to have a fully dried polymer. Nevertheless, drying commercial grades is a common procedure in the polymer transformation process. The higher polarity of PLA allows it to absorb more water than other polymers, such as polyolefins, and makes it more sensitive. 

Unexpectedly, the obtained specimens were different, although the same orientation, temperature, speed, and filling percentage were used. Filament specimens seemed to show higher porosities than pellet-based ones. However, FDM-obtained specimens were expected to show some porosity due to the spaces between beads. Thus, the pellet-based appearance was unexpected. Moreover, although the diameters of the nozzles were different, the filling percentage of the equipment was established to be 100%. Therefore, a similar porosity was expected but not attained. Additionally, defects could be expected with both methodologies but were not appreciable. 

To obtain the infill percentage, specimens were weighed and their mass was compared with that of a filled specimen. Table 1 shows the obtained results. The samples are named PLA (X-Y), where X indicates the layer orientation and Y the extrusion mechanism: F (filament) or P (pellet). 

**Figure 4:**
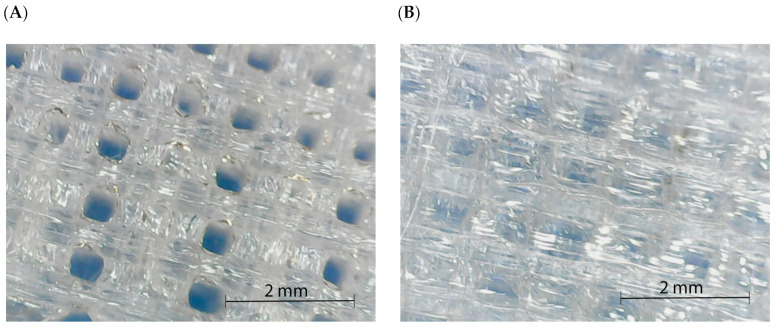
Printed structure of PLA 0/90 specimens produced through filament extruder (**A**) and pellet extruder (**B**).

The specimens obtained from the pellet extruder returned values that indicated filled or almost filled specimens. Moreover, porosity in the specimens was not observed as it appeared to be a compact material. The lack of porosity is depicted in Figure 4, which shows the structure of PLA0/90 specimens produced from the filament (A) and directly from the pellet (B). Although the pattern 0/90 of the layer deposition is observed for both specimens, the pellet specimen seems to have a compact structure. This compact structure could be due to higher retaining of the temperature, probably due to the melting process of the extruder, where a range of increasing temperatures is applied, while in the filament extruder, the temperature is applied mainly in the nozzle. Thus, PLA specimens from the pellet extruder have a lower viscosity during the deposition of the layer and a slower cooling process. Once the next layer is deposited, the higher viscosity of the already deposited layer, due to the higher temperature, enhances the interdiffusion of the polymer chains between layers and leads to better adhesion. Moreover, there was no squashing of the layers, as the thickness of both specimens showed almost the same value, 3.35 mm for the pellet and 3.33 mm for the filament. 

**Figure 5:**
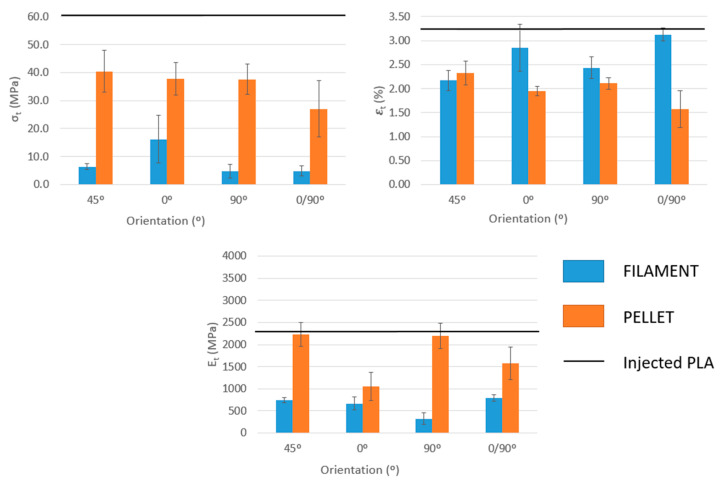
Tensile properties of the specimens obtained with different orientations of the beads and by direct pellet extrusion or filament deposition. PLA transformed through injection molding is used as a reference (PLA).

On the other hand, specimens obtained from PLA filament returned low infill percentages. It must be noted that the authors imposed a 100% filling strategy on the equipment. The infill percentage for the filament-based specimens varies significantly with the orientation of the beads. The infill percentage impacts the area of the section that opposes the tensile loads and affects the tensile properties of the specimens. Thus, specimens with lower infill percentages are expected to have lower tensile properties. Figure 5 shows the obtained values. 

The test data showed variations between the different sets of samples manufactured with both filament and pellet extrusion, with some sets showing large internal differences compared with PLA samples from injection molding. This effect could be related to the anisotropy of the printed structures. On the other hand, the reductions in mechanical properties were different depending on the orientations of the beads in the test specimens. The orientation of the layers causes significant differences in the mechanical resistance throughout the range of the printing orientation. The 0, 45°, and 90° orientations, manufactured by extrusion of pellets, are the most similar to PLA, which gives rise to mechanical resistance losses between 33% and 37%. The 0/90° orientation is the sample that achieved the lowest mechanical resistance, probably related to the different deposition of the layers resulting in higher porosity, although it was not estimated, in the structure. 

The most significant cases are the specimens manufactured through filament deposition, which results in mechanical strength losses ranging from 73% to 92%. This abrupt difference between both printing media can be related to the weak bond between layers or porosity between the layers. A post-treatment of the test specimens with dichloromethane or acetone, where the specimens are left in a sealed chamber for a finite time in an open container with the solvent, can increase the mechanical strength [23]. In this treatment, the chemical vapor melts the surface of the samples and fills the empty spaces of the sample, making it smooth.

The test specimens manufactured using pellet extrusion have higher tensile strengths than those produced by filament deposition. This fact is quite expected since the layers seem to present a better interfacial adhesion. However, in the group of pellet specimens, the results were unexpected since the trend should be the same as the filament specimens. In other words, the test specimens with an orientation of 0° should give the highest results since this is the orientation in which the stress of the test occurs. For the same reason, the specimens with an orientation of 90° reflect the results with more loss of tensile strength and the other orientations, 45° and 0/90°, have better resistance than those at 90° but are lower than those at 0°. In the case of 0/90°, as abovementioned, the porosity of the pattern probably affects the interlayer and probably is the reason for the reduced mechanical resistance. 

It should be noted that the number of perimeters of a sample also affects its strength. The perimeters are present in each layer and always align with the load axis. Therefore, the 45° and 90° layer orientations were not complete, as the perimeter had a 0° orientation.

Young’s modulus, which expresses the stiffness at the beginning of a tensile test, varies depending on the orientation of specimen layers (Figure 5). The cause for this effect could be related to the behavior of the individual filaments of the test specimens when subjected to tensile loads. In addition, differences can be observed depending on whether the sample is printed using filament or pellet extrusion. Test specimens manufactured by extruding pellets presented the orientations with higher values and, in some cases (45 and 90°), close to injected to PLA. On the other hand, in test specimens manufactured with filament deposition, losses from 66% to 86% were obtained for the orientation of 0/90° and 90°, respectively. Thus, pellet-printed PLA produced stiffer structures than filament-based specimens. The differences between Young’s moduli of the specimens when the printing strategy varies indicate that FDM-obtained samples are structures instead of materials. Young’s modulus is a fundamental property linked to a material and does not change with its shape but only when the structure of such material varies.

In these cases, the higher value of Young’s modulus, the more area is under the curve. This indicates the resilience of the material and, therefore, the test pieces with the layers oriented at 45° are the material that needs more energy to deform.

**Figure 6:**
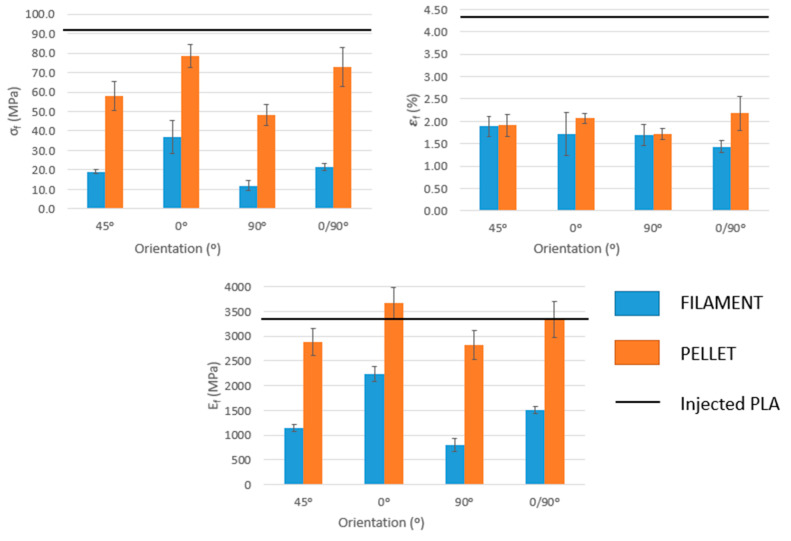
Flexural properties of the specimens that were obtained with different orientations of the beads and by pellet extrusion or filament deposition.

Finally, the strain at the break of the specimens was analyzed, and it was observed that filament or pellet-based specimens had an orthotropic behavior, in which the printing direction was significantly different from their behavior in the transverse direction. For example, PLA printed using filament shows an 18% higher strain at breaking in the axial direction compared to the transverse direction, and specimens printed using pellet extrusion show an 8% lower strain at breaking in the axial direction compared to the transverse direction. In the case of the pellet extruder, the orientations with a maximum strain at break are 45 and 90°. However, for 3D printing with filament, the maximum strain at break is achieved for orientations at 0/90° and 0°. It is worth noting the increase experienced by filament prints compared to pellet extrusion. Filament-printed samples are more deformable compared to pellet samples, probably because the increase in free volume around the polymer chains in the filament group favors their mobility, making their deformability higher. Due to its internal structure and the different tensile properties obtained for different bead orientations, such bead orientation has a noticeable impact on the mechanical properties of the specimens, increasing the interest in flexural properties (Figure 6).

A three-point bending test exerts forces on a body that tend to induce a tensile load in part of its cross-section and a compressive load in the remaining section. Flexural strengths are higher for the 0° orientation for both filament and pellet printing methods with values of 36.9 MPa and 78.9 MPa, respectively, which is in agreement with the tensile data. The orientations of 0°/90° and 45° are the next higher strengths, and the orientation of 90° has the least strength, 11.9 and 48.0 MPa. Therefore, the flexural test results show the same general trend as the tensile test results when pellet and filament materials are compared, as in Figure 5. Again, as in the tensile test, the strengths at the measured bending were much lower than the bending strength of PLA (92.1 MPa). This is probably because printed samples have weak interlayer bonding or interlayer porosity and are not compact specimens. For the same reason, as the bond between layers for printing with filament is weaker than for pellets, specimens returned lower values.

These flexural strength results further confirm that the orientation of the layers of the samples contributes to the anisotropy of their properties. This is probably caused by the directional processing of the 2D laminations. The resistance ratio between the highest (0°) and the lowest (90°) orientation is 1.6 for the pellet and up to 3 times for the filament. Instead, the resistance ratio between the highest orientation (0°) and the second highest (0/90°), for both cases, is 1.1.

In addition, Table 2 shows the performance according to the orientation of the layers and the printing method of the bending stresses at the breaking of the sample and the section of the sample. In the table, A_sp_ is the area of the section of the specimen obtained by FDM, A_i_ is the area of the section of a specimen obtained by injection molding, with a value of 0.42 cm^2^, σ_fi_ is its flexural strength (92.1 MPa ± 0.6), respectively.

The ideal case expected was the following:

(5) $$\frac{A_{sp}}{A_{i}}=\frac{\sigma_{f}}{\sigma_{fi}}$$

In this case, we can observe how the specimens manufactured with the pellet extruder have a closer correlation between the rupture sections and the stresses than the specimens manufactured with a filament. This yield ranges from 50 to 100% for the 0° samples. On the other hand, for the samples manufactured with filament, we can observe that no relationship follows, and as a consequence, yields of up to 9% are obtained, as is the case at 90°, which is the structure with the lowest mechanical properties.

Flexural modulus follows the same trend as Young’s modulus. Specimens printed using pellet extrusion with 0° orientation returned a flexural modulus 0.3 GPa higher than pure PLA. The results obtained for the flexural modulus and those for breaking loads clearly show that the specimen achieves higher mechanical strength when it is manufactured in the longitudinal direction of the stress axis. This result matches perfectly with those expected since, in the case of specimens manufactured through the deposition of filament, the lack of adhesion between the layers leads to breakage. For this reason, a considerable difference can be observed with those manufactured using the pellet extruder.

The maximum deformation follows the same trend as the flexural strength and modulus. Test specimens manufactured in the longitudinal direction to the load axis or the combination of directions in this axis and the transverse axis increase the maximum deformation. This is due to Hooke’s law, which states the linear relationship between elongation and stress for the elastic region.

**Figure 7:**
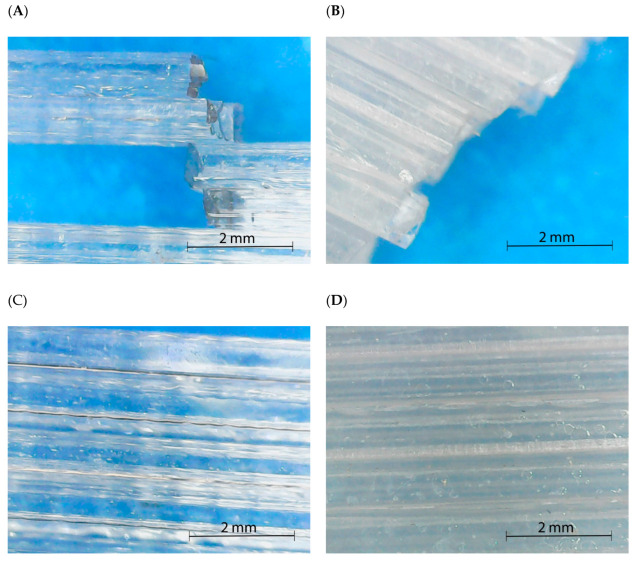
Optical microscope photography of the breaking area in flexural specimens orientated at 0° ((**A**) Filament; (**B**) Pellet) and structure overview at the same orientation ((**C**) Filament; (**D**) Pellet).

Specimens produced with the pellet extruder reported higher values than those observed for the filament due to the better adhesion of the layers. This better adhesion between layers, already shown in Figure 4, reported a different layer adhesion. Figure 7A,C shows the breaking point and the overview structure of flexural specimens orientated at 0° produced using filament, while Figure 7B,D are the same orientated flexural specimens but obtained using the pellet extruder. The observation was performed to assess the behavior of parallel layers. As in the 0/90° samples, in the filament specimens, the individualization of the filaments is observed at 0°. However, although the filament deposited could also be appreciated in the pellet specimens, there is no space between the filaments corresponding to an optimal interdiffusion of the polymer chains between layers [11]. This phenomenon was repeated in all the printed specimens with the pellet extruder in a single orientation. Moreover, another clear difference could be observed: while filament specimens tend to show a transparent behavior, pellet specimens are translucid or white.

**Figure 8:**
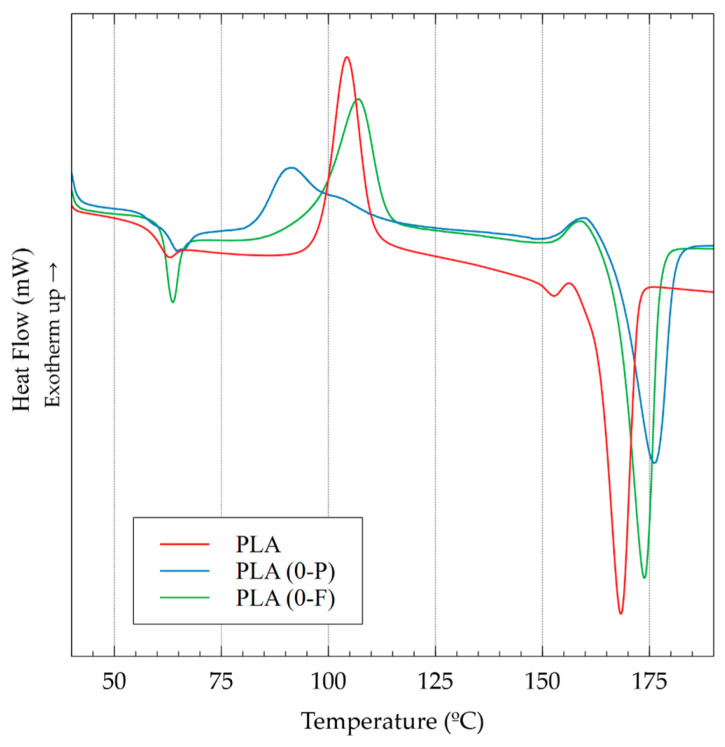
First melt of the flexural specimens at 0° and PLA injected samples as reference.

The different light interaction of both specimens is associated with a different crystallinity obtained by using a different cooling process during the printing. The significant differences appreciated in the specimens produced were quite unexpected, as the output temperature for both extruders was 200 °C and the bed temperature was kept at 55 °C, close to the T_g_ of PLA, to ensure a vitreous state in the deposited layer to enhance the adherence between layers. A first scan in the DSC was performed to evaluate differences in the cooling process (Figure 8).

The thermogram shows the thermal behavior obtained directly after the 3D printing of the specimens and a PLA from injection-molding transformation, used as a reference. Three different thermal transitions are appreciated for all the studied specimens: the T_g_, the cold crystallization (T_c_), and the melting process (T_m_). The T_g_ of the 3 types of processed specimens reported similar values (Table 3), but both 3D printed specimens showed slightly higher temperatures (1 °C) than PLA injected. The difference, however, is not representative. Moreover, the different behavior of the T_g_, with higher aging in the case of the PLA injected sample, is probably due to the different cooling processes of both transformation methodologies: in the injected sample, the temperature decreases to room temperature, while in the 3D printing process, the bed of the printer is kept at 55 °C during the printing process. The cold crystallization transition showed a similar behavior between PLA from the 3D filament and the injected sample, with a single peak at 106.9 and 110.8 °C, respectively. Nonetheless, PLA from the 3D pellet showed a broad peak at 90.9 °C with a shoulder at higher temperatures (102.9 °C). Moreover, the intensity of both transitions observed related to a cold crystallization is lower than that observed for the other samples. The enthalpy of the process is 23 J/g and 35 J/g for pellet and filament extruders, respectively. The broad peak with the shoulder indicates the production of two types of crystalline structures. However, as a unique melting process is observed in the sample, it could be related to the growth of crystals already in the sample and produced during cooling in the 3D printing process and the growth of other new crystals, where those already present are acting as nucleating agents. Thus, it will be in agreement with the single peak observed during the melting procedure with a slightly higher temperature than the other samples analyzed. Differences in the T_m_ are devoted to the different crystals obtained during the cooling down process and the heating performed in the DSC analysis. The crystals in the PLA from the pellets specimen required lower energy, as shown during the T_c_, while the new crystals growth in PLA from the pellet specimen and PLA from the injection sample required higher energy and presented a lower thermal stability. 

These differences in the crystalline behavior during cooling after the processing technology could be easily calculated as the crystallinity of the DSC samples that correspond to the crystallinity in the samples after the transformation process. This crystallinity is calculated as the difference between the normalized enthalpy (J/g of PLA) of the melting and the cold crystallization transitions and divided by the enthalpy of 100% crystalline samples of PLA [36]. The results showed a clear increment of the crystallinity in the pellet specimen from 3D printing, which is almost double the injected PLA and 43% higher than the filament specimen.

**Figure 9:**
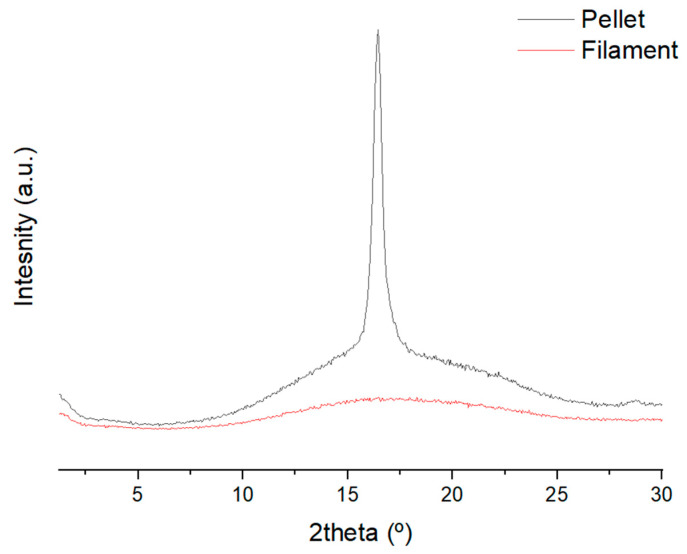
XRD difractograph of 3D printing specimens.

These results were corroborated with XRD, as the calculus of the initial crystallinity of the sample using DSC could have some errors due to the increasing temperature rate of the sample. Instead, XRD allows us to determine the crystallinity of the sample at a controlled temperature. The analysis was performed at room temperature directly from the specimens, and the results are shown in Figure 9.

The XRD difractograph showed an amorphous structure for PLA from the 3D filament, while a clear peak at 16.5° corresponding to PLA crystalline structure is observed for the pellet specimen [37]. The crystallinity index in XRD can be calculated from the ratio between the area corresponding to the crystalline part and the total area under the curve, which represents the area of the crystalline and the amorphous part [38]. The crystallinity index rendered a value of 27%, a value close to the one calculated using DSC. In addition to the differences in the crystallinity index calculated by both techniques, 3D printing with the pellet extruder confirms a slower cooling process that allows the PLA to crystallize in a higher quantity than 3D printing using filament. The slower cooling enhances the viscous state of the deposited layer and facilitates the adhesion between the layers, leading to lower porosities and higher mechanical properties. 

**Figure 10:**
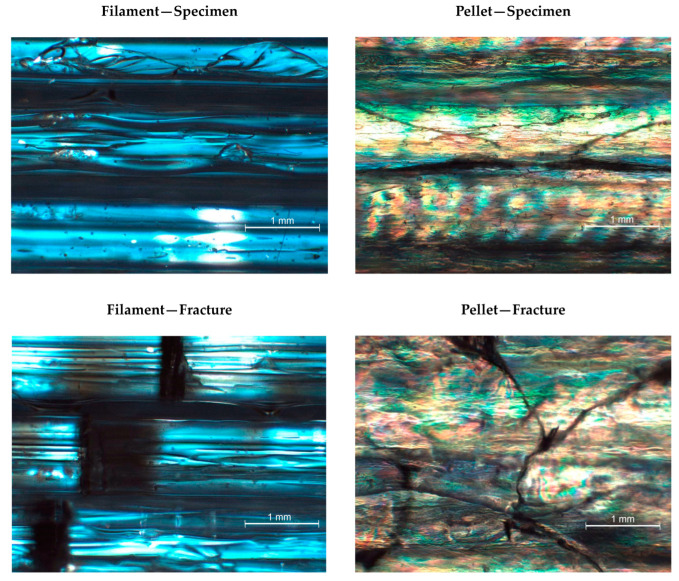
Optical polarized microscope images of the 3D specimens in broken and unbroken samples.

Polarized optical microscopy was performed to analyze any existent pattern in the crystallinity. The pictures were obtained from the same 3D flexural specimen orientated at 0°. Figure 10 shows the results for the 0° oriented samples for both 3D printing techniques in the material and a fracture region.

Again, the sample from the filament printing did not show any significant crystalline region, while the presence of the crystals in the pellet printing is apparent. Nevertheless, no apparent pattern in the crystals is observed in the pellet specimens. Regarding the fracture pictures, in the case of pellet printing, it is difficult to assess if there is some induced crystallinity in the region. There are some changes in the pattern in comparison with the unbroken specimen but not a clear tendency. In the case of filament printing, slightly induced crystallization is produced around the fracture, represented as the apparition of some yellow tonality.

**Figure 11:**
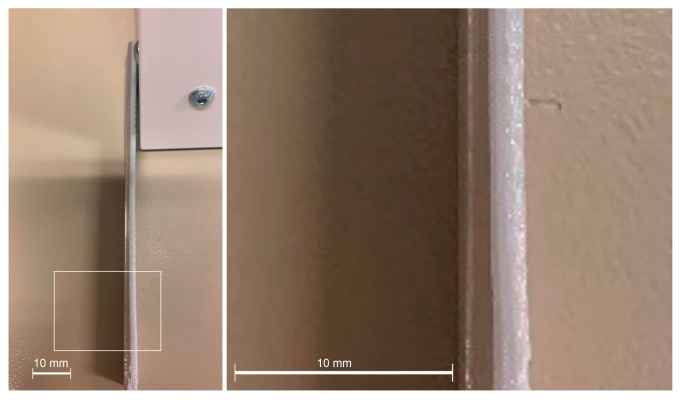
Two FDM technology specimens showing the areas manufactured using different PLA extruders in the FDM equipment.

Finally, specimens attained with a combination of pellet extrusion and filament deposition were flexural tested (Figure 11). The grey zone is made of filament, a colorant was used to differentiate them, and translucid/white by the pellet.

These test specimens were tested in two different configurations:

- PLA (0-BU);
- PLA (0-BD).

where BU are the bicomponent test specimens that have been tested with the part manufactured by pellets facing up; on the other hand, the BD test specimens had the pellet-based part facing down. Dual test specimens were manufactured only with an orientation of 0° since, in the previous tests, it was the orientation that provided better mechanical properties.

Table 4 shows the results of the bicomponent structures compared to the test specimens manufactured whole with filament or pellet.

The specimens that have a higher flexural strength and modulus are those with the part manufactured by pellet loaded at compression and the filament part loaded under tensile. The pellet-based section, when working under compression, shows more difficulty generating cracks than if it were working under tensile. However, the values are lower than the specimens fully manufactured with pellet extrusion. Otherwise, it should be noted that the maximum deformation in this test was given by the configuration in which the filament part works under tensile and the pellet-based part under compression. Nonetheless, due to the large deformation, it cannot be ruled out that when the deformations increase, both sections start working under tensile loads.

**Figure 12:**
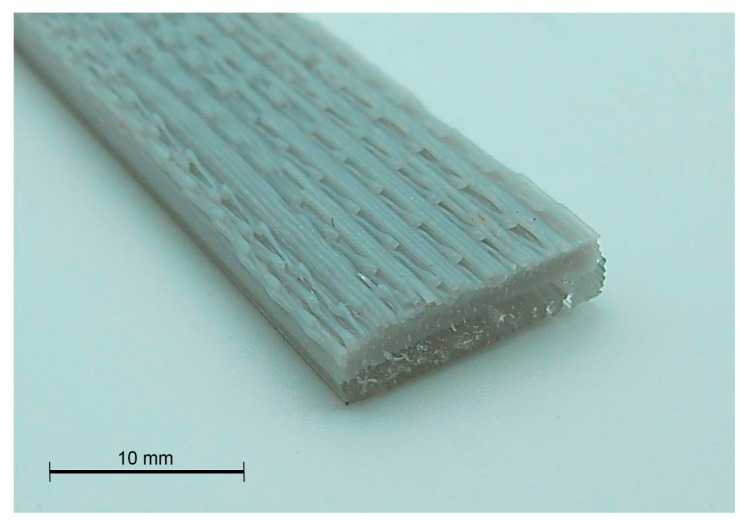
Fracture of the bicomponent specimen.

The strength ratio of a multi-component material (*R_s_*), used as a reference of the interface bonding, could be calculated as $R_{s}=\frac{\sigma_{Pellet/Filament}}{\sigma_{Filament}}$ where σ_Pellet/Filament_ is the strength of the multi-component material while σ_Filament_ is the strength of one of the filament pieces [11]. Using the filament as the component to relate, the R_s_ rendered in values of 1.72 and 1.43 for BU and BD, respectively. The expected strength of the material was expected to be the combination of both materials, as applying a simplified Rule of Mixtures (RoM) $\sigma_{Pellet/Filament}=\sigma_{Pellet}\cdot V_{Pellet}+\sigma_{Filament}\cdot V_{Filament}$. Considering half of the specimen was produced with each methodology, and a fulfilled sample has the volume of the 100% pellet specimen, the strength of the multi-component must be around 49.5 MPa. The strength value was higher than estimated in both cases, and the Rs was also superior to the theoretical one (1.34), which must be associated, despite the orientation of the testing, with an adequate interface between the layers of both methodologies. This optimal interface, obtained by the polymer diffusion related to the PLA from the pellet extruder that has a slower cooling process, facilitates the transmission of the stresses from one component to the other. The optimal transference of the stresses is demonstrated by the collapsing behavior of these samples, as neither component was debonded (Figure 12). 

## 4. Conclusions

The tensile and flexural properties of the specimens were noticeably affected by the internal structure of such specimens. Young’s moduli of the specimens, although it is a fundamental property of a material, vary with the changes in the bead orientation. Thus, FDM printed objects have to be analyzed as structures more than as materials. 

Nevertheless, pellet-based specimens returned tensile and flexural properties higher than filament-based specimens. The differences between both specimens were related to the different structures produced during the printing process. It was observed that pellet-based specimens had a higher infilling percentage than filament-based ones. A 100% filling percentage was, unexpectedly, obtained in the pellet-based specimens. Moreover, the higher infilling percentage seems to lead to a better interface between the layers. The combination of both technologies to obtain dual specimens did not increase the resistance or stiffness of pellet-based specimens. Nonetheless, the position of the layers had an impact on the deformation of the specimens. The Rs and the σ_P/F_ estimation of the dual samples showed an adequate stress transmission between both parts due to the good layer interface. 

The higher mechanical results shown in pellet-based specimens were due to the better interface between the layers. The melting process of the pellet, which was longer than the filament, resulted in a more viscous material with a slower cooling process. Thus, the adherence between layers is increased, and, as a consequence, the mechanical performance of these specimens is better in comparison with filament specimens. The slower cooling process was demonstrated by the increment of the crystallinity observed in DSC and XRD. Nonetheless, the polarized optical microscope did not show a clear pattern in the crystal growth. 

## Figures and Tables

**Table 1:** Infill percentages of the specimens that were obtained with different orientations of the beads and by direct pellet extrusion or filament deposition.

Material	Infill Percentage Tensile Specimens (%)	Infill Percentage Flexural Specimens (%)
PLA (45-F)	45 ± 3	42 ± 2
PLA (45-P)	100 ± 1	94 ± 3
PLA (0-F)	62 ± 5	49 ± 9
PLA (0-P)	100 ± 3	100 ± 1
PLA (90-F)	75 ± 7	65 ± 10
PLA (90-P)	100 ± 2	92 ± 6
PLA (0/90-F)	47 ± 3	34 ± 3
PLA (0/90-P)	100 ± 5	97 ± 4

**Table 2:** Deviations of the specimens from a filled sample and specific properties used to evaluate the performance of the specimens compared to a mold-injected sample.

Material	A_sp_ (cm^2^)	σ_f_ (MPa)	A_sp_/A_i_	σ_f_/σ_fi_	Performance (%)
PLA (45-F)	0.262 ± 0.002	19.1 ± 1.0	0.6	4.8	13
PLA (45-P)	0.418 ± 0.010	58.0 ± 7.5	1.0	1.6	63
PLA (0-F)	0.275 ± 0.017	78.6 ± 5.7	0.7	2.5	26
PLA (0-P)	0.498 ± 0.006	36.9 ± 8.6	1.2	1.2	100
PLA (90-F)	0.303 ± 0.023	11.9 ± 2.5	0.7	7.8	9
PLA (90-P)	0.410 ± 0.022	48.0 ± 5.4	1.0	1.9	50
PLA (0/90-F)	0.248 ± 0.005	21.3 ± 1.9	0.6	4.3	14
PLA (0/90-P)	0.413 ± 0.010	72.9 ± 10.2	1.0	1.3	78

**Table 3:** Thermal transitions of the first melting and the crystallinity of 3D printing specimens. PLA-injected samples are used as a reference.

Sample	T_g_ (°C)	T_c_ (°C)	T_m_ (°C)	Crystallinity after the Transformation Process (%)
PLA filament	61.6	106.9	173.4	14
PLA pellet	61.7	90.9	176.1	20
PLA injection	60.8	110.8	168.2	11

**Table 4:** Flexural properties of the dual FDM technology specimens and the impact of the position of the layers.

Material	σ_f_ (MPa)	ε_f_ (%)	E_f_ (MPa)
PLA(0-F)	36.9 ± 8.6	1.71 ± 0.48	2236 ± 149
PLA(0-P)	78.6 ± 5.7	2.07 ± 0.11	3664 ± 320
PLA(0-BU)	63.5 ± 5.2	3.55 ± 0.53	2423 ± 215
PLA(0-BD)	53.1 ± 5.5	2.36 ± 0.23	2052 ± 183

## Data Availability

Not applicable.
